# Effectiveness of Game-Based Learning Versus Problem-Based Learning Approaches in Teaching Microbiology to Phase II Medical Students

**DOI:** 10.7759/cureus.92330

**Published:** 2025-09-14

**Authors:** Shyamala R, Nidhin Jacob, Sana Basheer, Vidyalakshmi, Vathsalya MR

**Affiliations:** 1 Microbiology, Kodagu Institute of Medical Sciences, Madikeri, IND

**Keywords:** game-based learning (gbl), medical education, microbiology teaching, problem-based learning (pbl), student engagement and retention

## Abstract

Aim: The study aimed to evaluate the effectiveness of game-based learning (GBL) and problem-based learning (PBL) modules in teaching basic microbiology topics, examining their impact on student performance, retention, and overall student perception, while also identifying potential limitations to provide insights for future educational approaches in medical education.

Methodology: Two student groups participated in learning activities structured around four microbiology topics using GBL and PBL methodologies. Students' responses to these methods were collected, compared, and analyzed. Feedback was gathered to evaluate student perception and satisfaction. Additionally, a retention test was administered two weeks later to assess the durability of the knowledge acquired.

Results: Both GBL and PBL methodologies effectively improved student performance in microbiology, as indicated by mean scores, score ranges, and highest scores. However, statistical analysis showed no significant difference between the two methods, suggesting that GBL and PBL were equally effective. Student feedback indicated higher satisfaction and better overall perception of GBL than PBL, specifically in aspects such as understanding concepts, clearing doubts, and improving performance. Frequent application of these methods across various topics may further enhance student learning capabilities.

Conclusion: Incorporating GBL and PBL into medical curricula significantly enhances learning outcomes, promotes positive student perceptions, and fosters self-directed lifelong learning among undergraduate medical students. These innovative teaching methodologies could be beneficially integrated into medical education to enhance student engagement and educational effectiveness.

## Introduction

Microbiology deals with topics related to entities that are invisible to the naked eye. The majority of students are interested in natural objects or clinical conditions that can be seen with the naked eye. Nonetheless, microorganisms are key components of the biosphere, and a good microbiological background is required to understand bioburden in any clinical case [1].

Passive learning and lectures are still the common teaching formats. However, passive learning formats are no longer considered an adequate approach in most teaching situations. Instead, learning through a learner-centered approach by providing experimental experiences enhances learning, creates interest, and motivates students to learn better [2]. Students can be engaged with games as part of the teaching experience. Estoppey et al. evaluated the effectiveness of gamification by developing cards as teaching tools in learning microorganisms’ names, spelling, metabolisms, and critical analysis [1]. Myers’s study showed significant improvement in pre- and post-test scores among students involved in the game-based learning (GBL) model and problem-based learning (PBL) model. He noted that gamification should not be frequently used in academic programs, as it can become a less effective tool if overused [3].

If students are actively involved in the learning process, they can go beyond memorization and achieve a higher level of understanding and retention [4,5]. Problem-solving activities, active research, or group work improve active learning outcomes and foster student engagement [6-8]. These methods guide students to grasp knowledge and learning abilities through problem-solving activities and foster group discussion [9-11].

However, with changes in clinical demand and evolving societal expectations, there is a need to move towards innovative and integrated student-centric methods.

Hence, this educational study was conducted with the research question: Does the implementation of game-based and problem-based learning effectively teach microbiology to second-year medical students?

In this study, GBL and PBL were offered to phase II medical students on selected microbiology topics and assessed for multiple beneficial aspects such as applicability, engagement, motivation, learning, and acquisition of new skills.

## Materials and methods

This educational intervention study was conducted at the Department of Microbiology, Kodagu Institute of Medical Sciences, Madikeri, from September to December 2024. The study was conducted after obtaining institutional ethical clearance from the Institutional Ethics Committee of Kodagu Institute of Medical Sciences (Approval No: ECR/984/Inst/KA/2017, Ref: KoIMS/IEC/22/24-25, dated 28/08/2024).

After obtaining written informed consent, 30 students from the second-year MBBS program (Phase II in the Indian medical education system) were enrolled. Absentees and those not willing to participate in the study were excluded. Two study groups of 15 students each were formed. A briefing regarding the intervention was given to both groups. Faculty involved were prepared before the intervention [12,13]. Each session included the necessary resource materials.

Students were randomly allocated to groups using simple randomization (coin toss method). To account for differences in academic capability, baseline academic performance was assessed using previous microbiology test scores. No statistically significant difference was found between groups in baseline performance (p > 0.05), ensuring comparability.

Intervention

A small group discussion was planned, and one group was taught with a game-based module using three types of games, and the other with a problem-based module using problems and case scenarios. Four microbiology topics were selected to accommodate basic microbiology knowledge, recall learning, and clinical application [10]. The topics included type I hypersensitivity, cell-mediated immunity, sterilization and disinfection, and an overview of microbes. It was ensured that students were exposed to these topics for the first time and that they had not been taught during lecture classes.

Game-based module

Three different games were used for each topic as an intervention. The games included puzzles, crosswords, card games, word games, dumb charts, and treasure hunts. Tutors were involved in designing and conducting the game-based module. They were briefed about the significance of each game and the learning objectives for each topic. Depending on the type of game, the group was divided into five students each for convenience (Table 1).

**Table 1:** Detailed Description of the Educational Games Used

Topic	Game Type	Detailed Description	Learning Mechanism
Overview of microorganisms	Jigsaw Puzzle	Images related to bacteria, viruses, and parasites are cut into oddly shaped pieces. Students assemble to identify microorganisms	Visual pattern recognition and collaborative assembly
	Word Game	Students identify suitable terms after reading statements about microorganism definitions and classifications	Vocabulary reinforcement and concept matching
	Treasure Hunt	A series of clues related to topic locations. Teams solve clues to progress through microbiology concepts	Sequential learning through problem-solving
Cell-mediated immunity	Match the Word	Column A contains terms; Column B contains definitions. Students match corresponding pairs	Concept-definition association
	Crossword	Traditional crossword with microbiology terminology as answers	Memory recall and spelling reinforcement
	Puzzle	Similar to jigsaw, but focused on immunity cascade pathways	Sequential process understanding
Hypersensitivity Type I	Pick and Play	Students act out processes without speaking while teammates guess the mechanism	Kinesthetic learning and peer teaching
	Treasure Hunt	Progressive clues about anaphylaxis mechanism, detection, and treatment	Integrated clinical application
Sterilization and disinfection	Relay	Racing teams where each player completes one sequence step and passes to the next player	Sequential process learning with time pressure
	Musical Chairs	Chairs arranged in a circle, statements called out - students occupy chairs for correct statements only	Quick recall and decision-making
	Set in Order	Students arrange sterilization/disinfection methods in logical sequence	Process sequencing and prioritization

Problem-based module

In this module, problems and case scenarios were prepared. These problems were based on real-life situations. They were made interesting, motivating, and relevant to the medical profession. Each was prepared with conceptual objectives, keeping in mind the needs and interests of the students. The problems were kept open-ended, and students were allowed to think widely to come up with different solutions.

The same four topics that were used for GBL were used to conduct the PBL module. Students were sensitized to group dynamics, and the sessions were moderated by the principal investigator.

The seven-step PBL module of the Slovene Association of LSP Teachers was used to teach the PBL module [14]. The seven steps included: (1) clarification of unfamiliar terms, (2) problem definition, (3) brainstorming and hypothesis generation, (4) systematic arrangement of explanations, (5) formulation of learning objectives, (6) self-directed study, and (7) reporting back and synthesis of newly acquired information.

At the end of the sessions, the performance of the two groups was evaluated by peer-reviewed post-test questions with 10 MCQs to assess the level of understanding. Anonymous feedback on perception of learning was obtained using a structured questionnaire with a five-point Likert scale [15].

After two topics, a switch-over of the groups was done for the other topics so that both groups were exposed to both novel methods. A retention test was conducted two weeks after the study to determine the effectiveness of both methods using an MCQ test with 30 questions comprising all topics. After completion of the study, the gaming methods, problems, and results were shared with the rest of the class.

Method of data collection

Data were collected using MCQ-based tests after each session, along with a questionnaire for students' perception.

Data collection procedure

The data collection procedure included post-test scores, retention test scores, and responses to the questionnaire for perception.

Analysis

Post-test scores and retention test scores (mean, median, and standard deviation) were calculated. Statistical analysis was performed using the chi-square test for categorical variables and the independent t-test for continuous variables. A p-value < 0.05 was considered statistically significant. Data analysis was conducted using Microsoft Excel (Microsoft Corporation, Redmond, Washington).

## Results

This study was conducted to comprehensively analyze the impact of two learning methods using games and problems for teaching four selected microbiology topics. The study provided 60 total learning opportunities (15 students × 4 topics), with each student experiencing either three games or three problem-based scenarios per topic. Eight opportunities for learning were missed by students; hence, the total participation rate was 87%. At the end of each session, all 30 students took a post-test, and 28 students took a retention test during the study period.

Evaluation of learning efficiency

The learning efficiency of the two methods was evaluated by post-test scores. As shown in Table 2, the mean scores for GBL and PBL in the first topic “Overview of Microorganisms” were 8.1 and 7.7, respectively; for the second topic “Cell-Mediated Immunity” they were 5.1 and 7.2; for the third topic “Hypersensitivity” they were 7.0 and 7.5; and for the fourth topic “Sterilization and Disinfection” they were 6.9 and 7.8. The median scores for each topic were 8, 6, 7, and 8 for GBL, and 8, 7, 7, and 6.6 for PBL.

**Table 2:** Educational Games and Activities for Microbiology Learning Objectives

S. No.	Topic	Games Used	Learning Objective
1	Overview of microorganisms	Jigsaw puzzle, word game, treasure hunt	To identify the bacteria, viruses, parasites, and fungi
2	Cell-mediated immunity	Match the word, crossword, puzzle	Activation and propagation of cell-mediated immunity
3	Hypersensitivity Type I	Puzzle, pick and play, treasure hunt,	Mechanism, detection, and treatment of anaphylaxis
4	Sterilization and disinfection	Relay, musical chair, set in order	Types of sterilization and disinfection methods

The range of post-test scores varied from 5 to 10 when comparing both methods. However, when differentiated by topics, the range with the highest marks was seen in GBL. Statistical analysis showed no significant difference between GBL and PBL methods overall (p = 0.3, SD = 1.6) or for individual topics. The retention test showed a mean score of 17 out of 30, with a median of 16 and a score range of 9-22.

Evaluation of learning satisfaction

**Figure 1:**
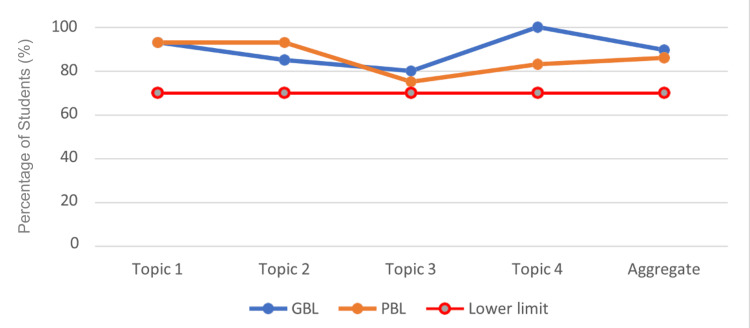
Perception of whether teaching methods could be used effectively for other subjects or topics. GBL was viewed as more adaptable and applicable across subjects, as reflected in the higher percentage of positive responses. GBL: game-based learning; PBL: problem-based learning.

Feedback on the perception of the two novel methods was evaluated after each session. The gaming group gave the highest scores for GBL module usefulness, found it interesting, reported that it helped understanding, and noted that it could be used to study other topics as well (Figure 1). Students from the gaming group reported higher satisfaction rates, indicating that GBL promoted self-directed learning, helped with doubt clearance, improved performance, aided retention, and was suggested for more frequent usage, as shown in Table 2. The satisfaction rate for the second topic was less than 70% for its usage, frequency, clearing doubts, and effectiveness in learning by the gaming method.

**Table 3:** Evaluation Criteria and Indicators for Assessing Student Satisfaction and Learning Effectiveness

Evaluation Question	Indicators
How satisfied were the students with the teaching?	% of students satisfied (>70%)
How effectively did the learning methods help them learning?	% students involved in gaming & discussion. Post test Mean Score >6, P<0.001

**Figure 2:**
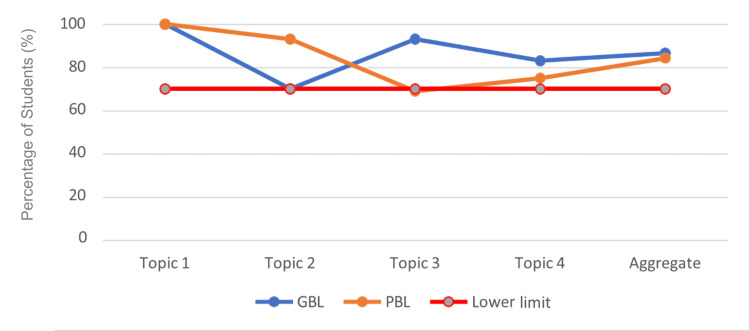
Perception of whether the teaching methods promoted SDL. While both methods received high marks, GBL maintained a slight edge over PBL, reinforcing its role in encouraging independent learning. GBL: game-based learning; PBL: problem-based learning; SDL: self-directed learning.

**Figure 3:**
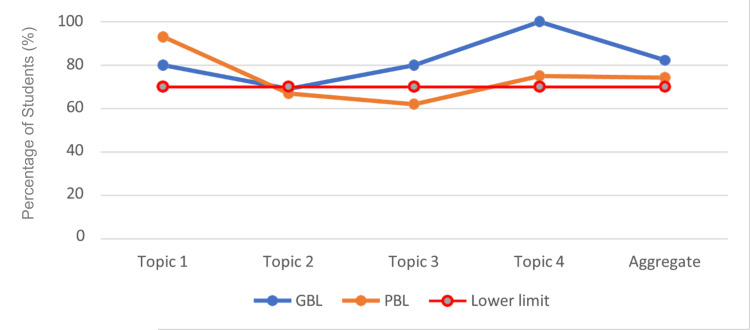
Perception of how well each method helped students in clearing doubts. GBL again received higher ratings, particularly in Topic 4, suggesting it facilitated better clarification of concepts than PBL. GBL: game-based learning; PBL: problem-based learning.

Students felt that PBL was effective in learning microbiology topics. A satisfaction rate greater than 70% was achieved with respect to making the topic interesting, promoting better understanding, encouraging self-directed learning, helping clear doubts, improving retention, and enhancing performance (Figure 2). However, the satisfaction rate did not meet the set criteria in the third topic. Students felt that PBL was not helpful and did not aid in doubt clearance for hypersensitivity topics (Figure 3).

**Figure 4:**
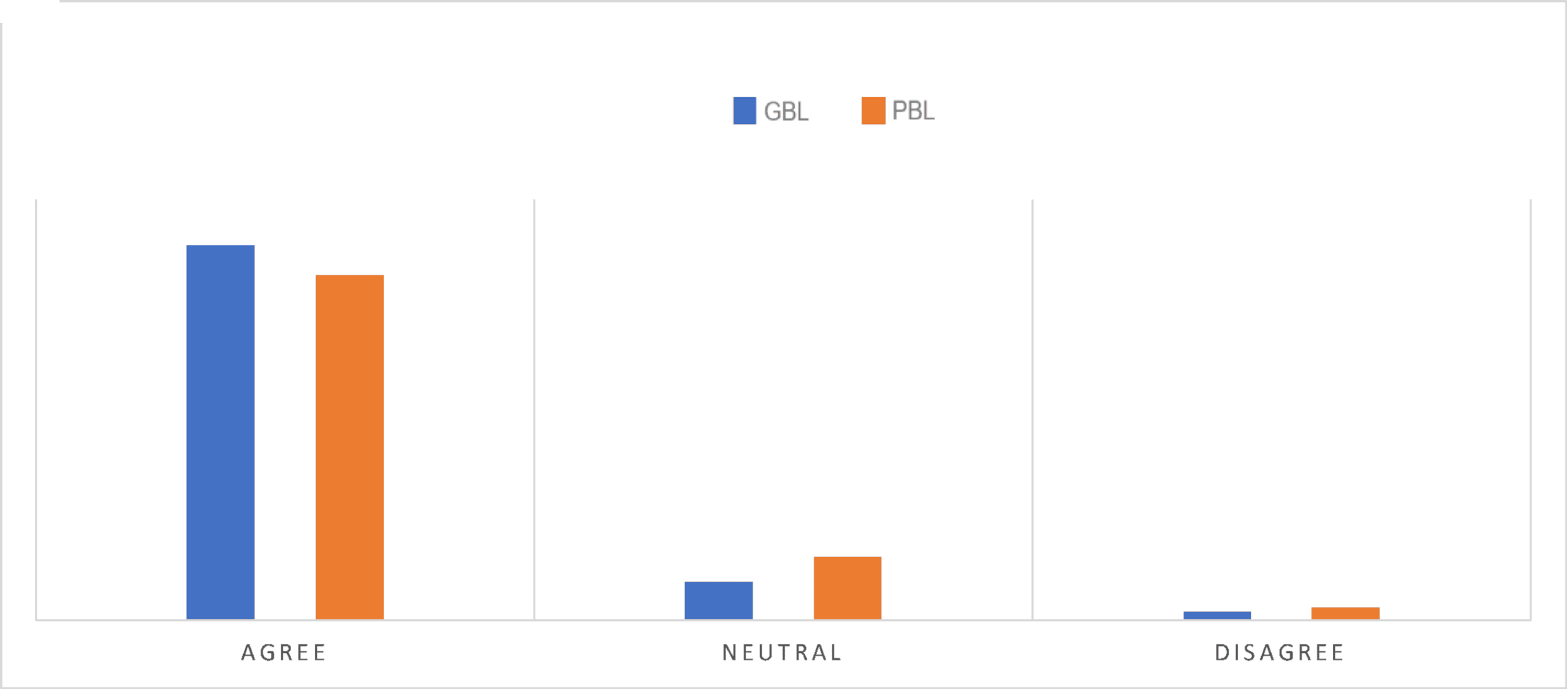
Comparison of student satisfaction levels between game-based learning (GBL) and problem-based learning (PBL). The x-axis represents levels of agreement (agree, neutral, disagree), and the y-axis shows the number of responses. A higher proportion of students agreed with the effectiveness of GBL (89%) compared to PBL (82%).

The overall satisfaction with GBL and PBL was 89% and 82%, respectively, where the set criteria were >70% (Figure 4).

**Figure 5:**
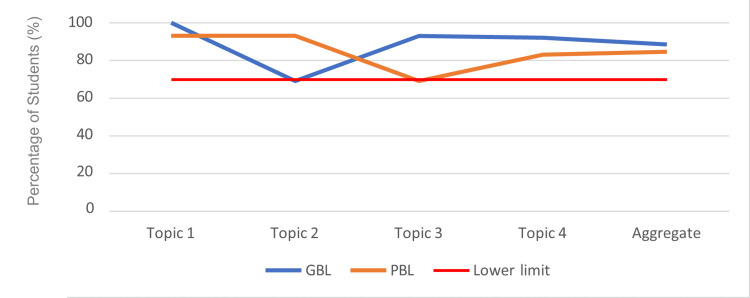
Student perception of the general usage of GBL and PBL across four microbiology topics. GBL consistently received higher usage ratings than PBL, particularly in the first and fourth topics. Aggregate values also showed a stronger student preference for GBL. GBL: game-based learning; PBL: problem-based learning.

**Figure 6:**
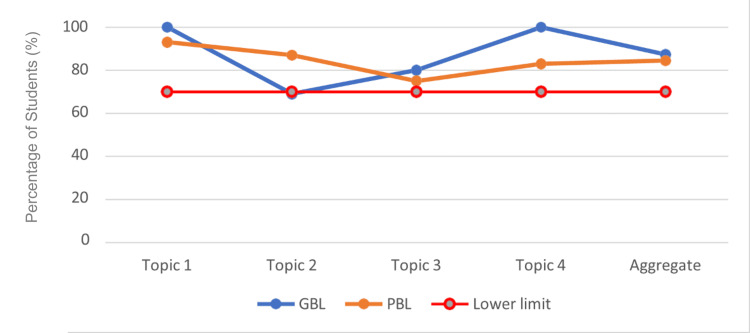
Student perception of the overall effectiveness of GBL and PBL in learning microbiology. While both methods were considered effective, GBL maintained a marginally higher effectiveness score across all topics and in the aggregate GBL: game-based learning; PBL: problem-based learning.

**Figure 7:**
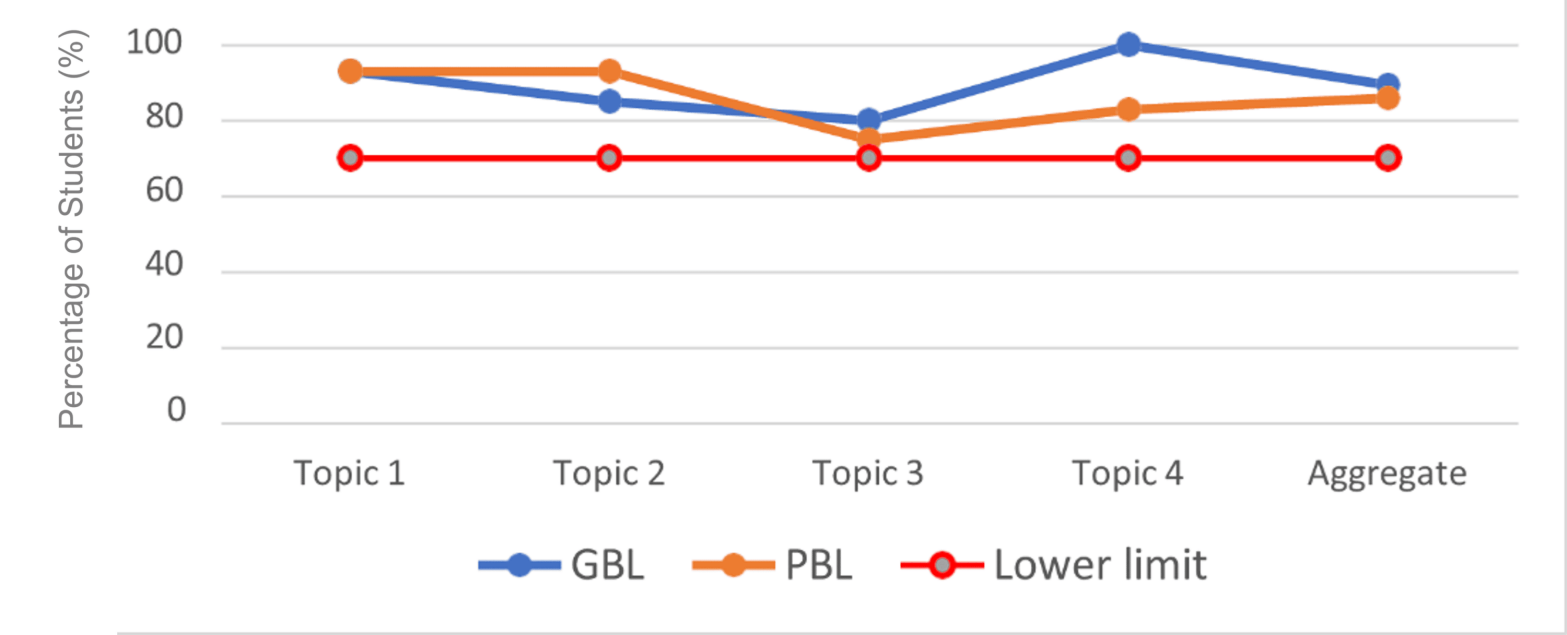
Comparative interest levels of students toward both learning methods. GBL received notably higher interest percentages across all topics, especially for Topic 1 and Topic 4, suggesting it was more engaging for learners. GBL: game-based learning.

**Figure 8:**
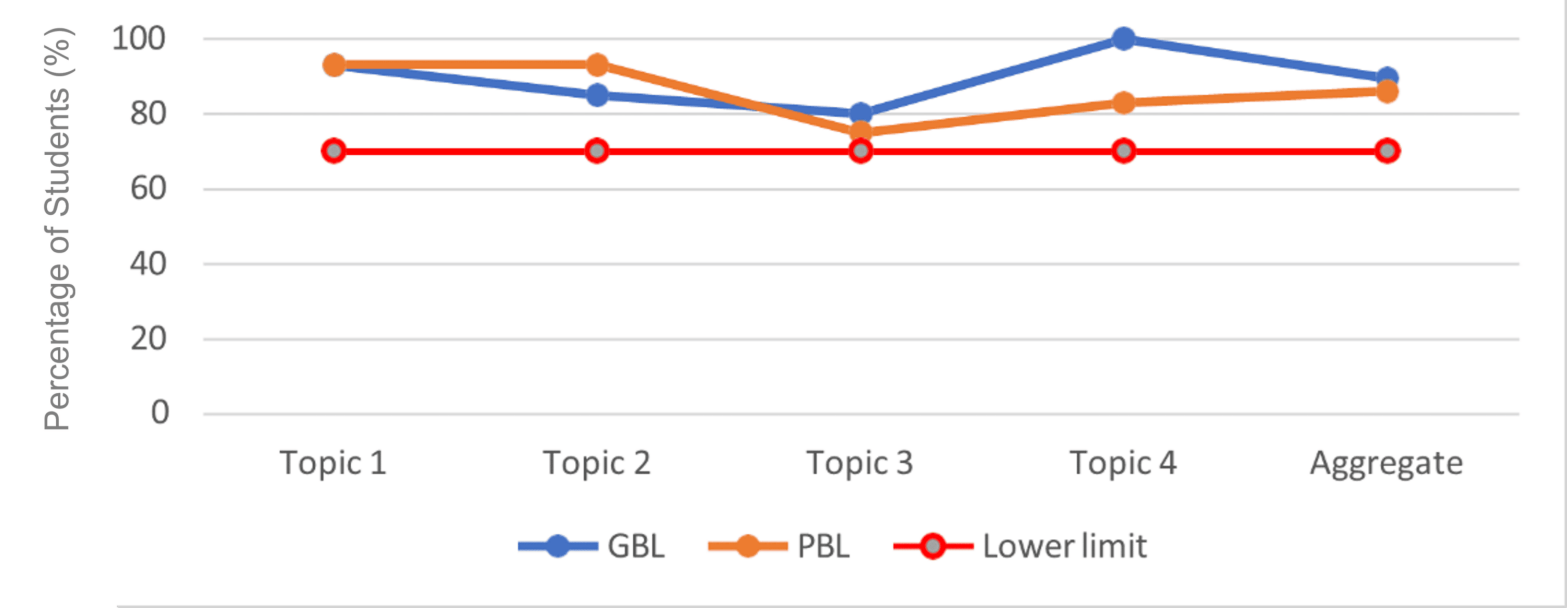
Student ratings of how the two learning methods aided conceptual understanding. GBL outperformed PBL, with scores surpassing 90% in some topics, notably Topic 1 and Topic 4. GBL: game-based learning; PBL: problem-based learning.

**Figure 9:**
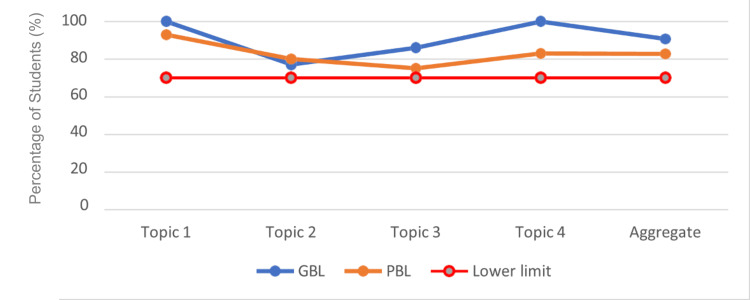
Student feedback on whether the learning method improved academic performance. GBL consistently had higher ratings, suggesting students found it more impactful in helping them perform better in tests and assessments. GBL: game-based learning; PBL: problem-based learning.

**Figure 10:**
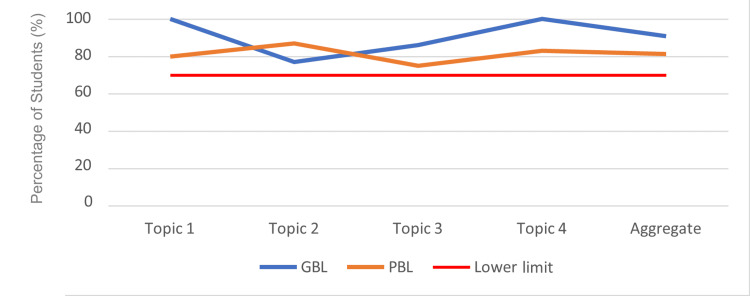
Student ratings of how the two methods aided retention of knowledge. GBL scored notably higher across all topics, indicating it was more effective for long-term memory. GBL: game-based learning; PBL: problem-based learning.

**Figure 11:**
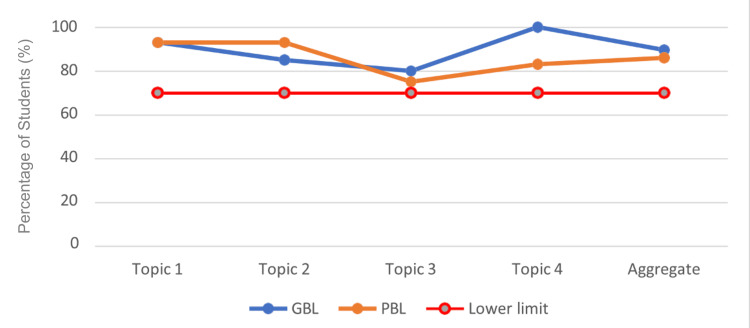
Student opinions on how frequently each method should be used in future sessions. GBL was favored for more frequent use, highlighting its popularity and perceived usefulness among students. GBL: game-based learning; PBL: problem-based learning.

The students’ perception of the use of GBL versus PBL modules in learning microbiology was 89% and 86%, respectively (Figure 5). When comparing the effectiveness and beneficence of the two methods, students felt that games were more effective (88%) than PBL (85%) (Figure 6). Students felt that the game-based module was more interesting (95%) compared to PBL (86%) (Figure 7). They perceived that games helped them understand the facts better (95%) than PBL (79%) (Figure 8). Students perceived that games helped with doubt clearance (85%), retention (91%), and improved performance (91%) when compared to PBL methods (75%, 81%, 83%) (Figures 9 and 10). Students opined that games should be used more frequently (89%) than problem-solving activities (80%) (Figure 11). An aggregate of four topics across all feedback showed that the game-based module was more satisfactory than problem-based learning.

Students remained neutral on the overall perception of GBL (9%) and PBL (14%). Disagreement rate on these modules was (1.5%, 3%) on GBL and PBL, respectively.

## Discussion

The new CBME novel methods of teaching, like GBL and PBL modules, have a unique advantage in creating an atmosphere of relaxed, stress-free, and discussion-friendly environment. By implementing these novel methods, students become independent by searching resource materials, improving networking, and establishing communication with teammates [9]. Hence, students develop the ability to review literature, prioritize self-directed learning, teamwork, and language expression. With these, students get more time to refine their contributions, which are generally thoughtful compared to passive learning. This method provides insight into the facilitators of feasible educational strategies that can contribute to the reformation of didactic activities [10]. Hence, these novel methods are used to supplement regular teaching, aiming to enhance student engagement and confidence.

In this study, learning was student-centric and involved fun-filled activities. The mean score of the GBL was 6.8/10, and the median was 7.2/10. However, the mean score varied for each topic. This could be explained by the facilitator factor or the type of game executed. The maximum score was achieved in the gaming method. Students found difficulty in the posttest on the second topic, cell-mediated immunity, which had new terminology and the sequential cascade of steps. In this case, posttests involved memory recall and accuracy of steps. This showed that one exposure is not sufficient to retain certain facts that are new, crucial, and require recall [5]. There was no statistical difference between the topics taught using the GBL module. This explains that uniformity and novelty were maintained in teaching and learning through games.

In the present study, the PBL group achieved a mean of 7.2/10 and a median of 7.5/10 with SD 0.8, which was a slightly higher score compared to the gaming method. But the highest score attained by the PBL method was lower compared to the GBL method. There was no statistical difference between the different topics taught using this method. There was consistent performance by the students for all four topics, which could be explained because the facilitator involved was a senior faculty cum principal investigator who could moderate the specific learning objectives crucially [11].

This study shows that microbiology may be taught using both GBL and PBL modules. But there was no discernible statistically significant difference between the two approaches. These results imply that both GBL and PBL are appropriate and advantageous for teaching microbiology subjects [10,13]. However, the conceptual goals of each subject must be closely matched with the way games and challenges are created and executed.

During the study period, students were thrilled to participate in GBL and PBL and felt more motivated to learn than in the regular didactic small group discussion (SGD) activity. The feedback on the perception of GBL was better than the PBL module. Though problem-solving activities could make students engage in discussion and mindfulness, students liked to be actively moving, talking, and having fun. They felt GBL is more fun learning than facilitated discussions [7,8]. In this study, the overall student perception of GBL was more favorable than that of PBL in terms of enhancing understanding, clarifying doubts, and improving academic performance. The findings suggest that frequent implementation of these methods across various topics can enhance learning abilities and knowledge retention. The current study used online resource sharing, MCQ tests, and feedback collection, which motivated students to use the digital mode in their curricula.

Both these modules are facilitator-dependent. Usage of these modules is dependent on the facilitator's steering. The group dynamics are controlled by the facilitator. Participation and trainer-learner and learner-learner interactions are pivotal for the success of these modules [11,14]. Therefore, it is essential that the trainer is well-versed in each subject and how it could be used in interesting games and difficulties. Additionally, the trainer should be able to monitor group interactions, directing them toward the learning goals while permitting unstructured conversation within the allocated time.

Imwattana's study showed that, in contrast to a control group that did not play games, medical microbiology results were considerably enhanced by gaming [6]. According to our study's findings, there was a statistically significant improvement in the topic of antimicrobial resistance, but no significant differences were detected in the other three themes. Gaming group members gave the BactoBattle game high marks, scoring it 3.8 out of 5 for fun and a 3.7 out of 5 for its influence on learning motivation. BactoBattle is a digital card-based game designed for medical microbiology learning, focusing on bacterial identification and antimicrobial resistance concepts. However, neither the motivation scores (P = 0.562) nor the overall learning enjoyment (P = 0.441) differed significantly between the player and non-player groups. A significant number of players (11; 91.7%) indicated that they would be willing to keep playing the game and suggest it to their peers, which is also the attitude that our study participants exhibited.

Beylefeld and Struwig conducted studies on gaming methods on various microbiology topics and found that the overall result was pairing students' perceived emotional states to succinct dimensions of flow [5]. Four dichotomous pairs of response categories emerged in their study: pleasant preoccupation versus boredom (transformation), enjoyable stimulation versus fear of failure (loss of self-consciousness), focused commitment versus a disturbed balance between challenge and skills (sense of control), and self-knowledge versus rigid predisposition (receiving unambiguous). During the conduct of our study, facilitators were made aware of the nature of the qualitative outcome and asked to coordinate the positive nature of the group.

In Baviskar's study, crosswords, quizzes, and scenarios were used in community medicine topics, and it was found that a total of 237 (59.25%) students reported that they found online lectures tiresome at times, and a total of 364 (91%) students stated that GBL helped them study better [7]. Male students showed significantly better responses to gamification as compared to online lectures. In his study, 94% of students responded that they would like similar content on all subjects. Our study showed that 89% of students want to use this method for other topics as well.

According to a study on the PBL module by Prajapati et al., students in the PBL group significantly outperformed those in the standard teaching group in terms of mean scores [10]. Their results showed that 91% of students thought PBL improved their ability to study on their own, and 100% of students thought it helped explain basic microbiology concepts. By contrast, 84% of students in our survey concurred that the PBL modules encouraged self-directed learning. Similarly, Sun’s research on the PBL technique showed that it had a favorable impact on several aspects of student learning [9]. In that study, 83.76% of students reported improvement in literature retrieval, 74.79% reported better independent study skills, more than 60% reported improvement in language expression and teamwork, and 53.85% reported improvement in critical thinking. Our study’s results closely match these conclusions, demonstrating how well the PBL approach fosters a variety of learning competencies.

According to Bailey and Smith, student perceptions of the PBL approach can be categorized into three themes: (1) understanding the true importance of each concept, (2) acquiring the required knowledge, and (3) becoming familiar with the process of change in teamwork [12]. In the present study, these key aspects of the PBL module were also addressed, aiming to foster familiarity with collaborative learning and promote self-directed engagement.

This study has several limitations. First, the sample size was relatively small (n = 30), limiting generalizability. Second, we could not determine which specific games had a greater impact on learning due to the combined approach. Third, the study design did not assess these methods as supplements to traditional lectures, which limits practical implementation guidance. Fourth, the retention test was conducted only once at two weeks, and longer-term retention was not assessed. Fifth, inter-rater reliability for facilitators was not measured, potentially introducing bias in delivery quality. Additionally, there was a lack of thorough and organized information acquisition, insufficiently focused themes, and rambling conversations [11,13]. Even when more structured, traditional teaching approaches frequently fail to engage students effectively and restrict their initiative. Similarly, overly simple or uninteresting game designs can cause GBL to neglect important content, grow monotonous over time, or fail to maintain learner attention [5,8]. Both PBL and GBL offer unique benefits and drawbacks. As a result, a blended strategy that carefully incorporates and adapts components from both approaches may provide a more successful and balanced educational experience [4,14].

## Conclusions

This study concludes that GBL and PBL modules have a positive impact on the learning of microbiology. Supplementation of formal lectures with informal learning approaches helps counter feelings of despair related to assimilating the vast factual content of medical microbiology. It also emphasizes that gamification and problem-solving activities involve multiple domains of learning. These novel methods help improve medical students’ performance in microbiology.
